# A close examination of BCRP's role in lactation and methods for predicting drug distribution into milk

**DOI:** 10.1002/psp4.13243

**Published:** 2024-09-18

**Authors:** Caroline Sychterz, Hong Shen, Yueping Zhang, Michael Sinz, Amin Rostami‐Hodjegan, Brian J. Schmidt, Lu Gaohua, Aleksandra Galetin

**Affiliations:** ^1^ Division of Pharmacy and Optometry, Centre for Applied Pharmacokinetic Research, School of Health Sciences University of Manchester Manchester UK; ^2^ Bristol Myers Squibb Princeton New Jersey USA; ^3^ Certara Predictive Technologies, Certara UK Sheffield UK

## Abstract

Breastfeeding is the most complete nutritional method of feeding infants, but several impediments affect the decision to breastfeed, including questions of drug safety for medications needed during lactation. Despite recent FDA guidance, few labels provide clear dosing advice during lactation. Physiologically based pharmacokinetic modeling (PBPK) is well suited to mechanistically explore pharmacokinetics and dosing paradigms to fill gaps in the absence of extensive clinical studies and complement existing real‐world data. For lactation‐focused PBPK (Lact‐PBPK) models, information on system parameters (e.g., expression of drug transporters in mammary epithelial cells) is sparse. The breast cancer resistance protein (BCRP) is expressed on the apical side of mammary epithelial cells where it actively transports drugs/substrates into milk (reported milk: plasma ratios range from 2 to 20). A critical review of BCRP and its role in lactation was conducted. Longitudinal changes in BCRP mRNA expression have been identified in women with a maximum reached around 5 months postpartum. Limited data are available on the ontogeny of BCRP in infant intestine; however, data indicate lower BCRP abundance in infants compared to adults. Current status of incorporation of drug transporter information in Lact‐PBPK models to predict active secretion of drugs into breast milk and consequential exposure of breast‐fed infants is discussed. In addition, this review highlights novel clinical tools for evaluation of BCRP activity, namely a potential non‐invasive BCRP biomarker (riboflavin) and liquid biopsy that could be used to quantitatively elucidate the role of this transporter without the need for administration of drugs and to inform Lact‐PBPK models.

## INTRODUCTION

The benefits of breastfeeding are well known, but the decision to breastfeed is impacted by several factors: from personal to societal expectations, physical (i.e., the mother's ability to breastfeed) and medical (i.e., medications needed over the lactation period).^1^ Of those potential impediments, the most practical one to navigate should be deciding to breastfeed while taking a medication. However, few drug labels provide human‐specific, definitive dosing advice despite the implementation (and best intentions) of the Pregnancy and Lactation Labeling Rule implemented by the FDA in 2015.^2^ As with other special populations, like pediatrics and pregnancy, the ethical landscape and risk‐adverse nature of industry means that clinical lactation studies are often rare.^3^ From a safety perspective, it is simple enough to contraindicate any drug during lactation, but in many cases that approach is not practical as lactating women have medical needs as well.^4^ Moreover, these typically lead to “off‐label” use of drugs as it is shown in the case of drug use in neonates.^5^ Another risk, often over‐shadowed by safety, is that of withholding a safe drug from someone who could benefit from it.

The attention of industry, academia, and regulatory agencies has focused on the gap in properly informing clinicians on the safe use of drugs during lactation in recent years. While still only a small portion of all post‐marketing commitment (PMC) requests from the FDA, the number of lactation‐focused PMCs by the agency has increased over time.^6^ In‐hand with regulatory agency interest, industry, and academia have followed suit with the relatively recent formation of large collaborations like the ConcePTION, MPRINT, and BEGIN projects (ConcePTION (imi‐conception.eu); MPRINT Hub: Indiana University; Breastmilk Ecology: Genesis of Infant Nutrition (BEGIN) Project|NICHD ‐ Eunice Kennedy Shriver National Institute of Child Health and Human Development (nih.gov)), all with the intent to provide a stronger knowledge base around the dynamics of lactation and recommendations for informing mothers and their doctors when it comes to lactation and concurrent use of medicines.

With clinical data around drug distribution into milk and subsequently the infant being absent in the worst case, or available but representing small and sparse datasets in better cases, an opportunity exists for quantitative prediction methods to potentially fill existing information gaps.^7^ Specifically, quantitative assessment using physiologically based pharmacokinetic (PBPK) modeling has proven to be incredibly useful in predicting metabolism and transporter‐mediated drug–drug interactions, food effects and recently also pharmacokinetics in special populations.^8^, ^9^ While PBPK modeling of lactation is still a relatively young application, this approach has the potential to be as predictive and impactful in informing drug labels as it has been in the aforementioned areas. Interest in lactation PBPK modeling (Lact‐PBPK) has increased as evidenced by the general increase in the publication of Lact‐PBPK models in literature over time.^10^, ^11^ As with application of PBPK modeling in other areas, Lact‐PBPK is an evolving science with opportunities for improvement and better implementation in drug development to assess lactation‐based risks (and drug labels) early.

An important parameter implemented in perfusion‐based Lact‐PBPK modeling is the milk:plasma (M:P) ratio, which is the ratio of drug concentration in milk versus plasma. One of the many challenges with a M:P ratio, or other ratios commonly used in PBPK modeling (like the blood:plasma or tissue:plasma ratios), is that it is a static value used to describe the dynamic event of drug distribution into not only the mammary gland, but also milk through the mammary gland. As mentioned previously, clinical lactation studies are rare during drug development leaving modelers without a measured human M:P ratio to implement in a Lact‐PBPK model. In some instances, M:P ratios are available from preclinical species; however, the concern with using a M:P ratio from animals is its relevance to human. Besides the obvious differences in terms of number of offspring and duration of the lactation period, milk composition is different between mammalian species with varying concentrations of fat and protein in human versus the typical species used in preclinical development.^12^ Estimating the amount of drug excreted into human milk is not a new concept with prediction methods dating back to the mid‐1980's where multiple authors (e.g., Fleishaker, Atkinson & Begg) used drugs' physicochemical properties and milk composition to predict drug distribution into milk.^13^, ^14^ It is important to note that these equations predict M:P ratios based upon steady‐state conditions of unbound drug distribution between milk and plasma via diffusion in the absence of active transport. Distribution predictions become more complicated if a drug is a substrate of a transporter, and recent equations have made attempts to consider active efflux of drugs by incorporating in vitro efflux ratios from permeability assays.^15^, ^16^ Examples of M:P ratios predicted by passive permeability and by including active transport are in Table 2 with equation details in Table S4.

**FIGURE 1:**
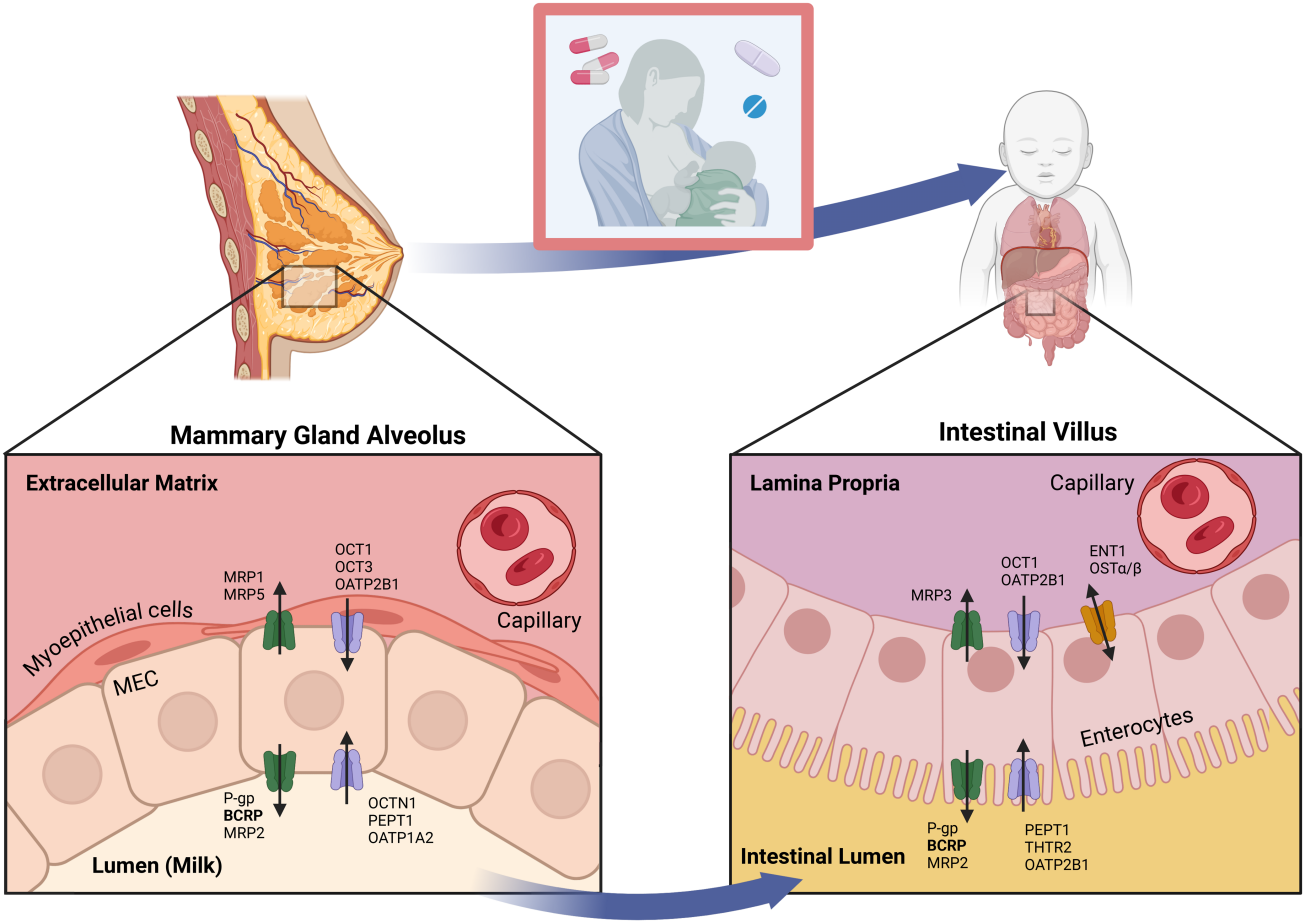
Drug transporters expressed in human mammary epithelial cells and comparison to clinically important transporters expressed in enterocytes. Transporters listed in mammary epithelial cells are not inclusive and represent mRNA and/or protein expression.^18^ Clinically important transporters in the intestinal villus have been adapted from Galetin et al.^69^ BCRP, breast cancer resistance protein; ENT1, equilibrative nucleoside transporter 1; MRP1‐3,5, multidrug resistance‐associated protein 1‐3,5; OATP1A2, organic anion transporting polypeptide 1A2; OATP2B1, organic anion transporting polypeptide 2B1; OCT1/3, organic cation transporter 1/3; OCTN, organic cation transporter novel 1; OSTα/β, organic solute transporter alpha/beta; PEPT1, peptide transporter 1; P‐gp, P‐glycoprotein; THTR2, thiamine transporter 2. Figure created in BioRender (www.biorender.com).

A wide range of drug transporters have been identified in mammary tissue either at mRNA and/or protein levels (Figure 1), including organic cation transporters (OCT), organic anion transporting polypeptide (OATP) and those from the ATP‐binding cassette superfamily, for example, breast cancer resistance protein (BCRP, *ABCG2*) and P‐glycoprotein (P‐gp, *ABCB1*).^17^ The exact membrane has yet to be confirmed for several of these transporters, although based on their location in other tissues, many transporters identified actively uptake substrates into mammary cells either from the apical (e.g., OCTN1/2 (*SLC22A4/5*), PEPT1 (*SLC15A1*)) or basolateral side (e.g., OCT1/3 (*SLC22A1/3*), OATP2B1 (*SLCO2B1*)), while only a few transporters efflux substrates from the mammary cells into milk (e.g., BCRP and P‐gp).^18^ Of particular interest from a drug development and safety perspective is the BCRP transporter, which has been identified on the apical membrane of mammary epithelial cells.^19^ While BCRP's role is seen as protective at many membrane interfaces (brain, liver, and intestine), its location in breast tissue highlights its role in transporting endogenous substances, such as riboflavin, bile acids, and steroid metabolites,^17^ and poses the risk of active transport of drug substances into milk, facilitating drug exposure to infants.

The purpose of this review is threefold: (1) to highlight what is known about BCRP in mammary tissue, (2) evaluate current quantitative methods of measuring and predicting BCRP's role in lactational drug distribution, and (3) discuss methods to address gaps in existing knowledge from a quantitative perspective to allow better and earlier quantitative predictions of drug distribution into milk and associated risk during lactation.

## PRESENCE OF BCRP IN MAMMARY TISSUE AND ITS ROLE DURING LACTATION

### In vitro cellular data of BCRP in mammary tissue

The presence of transporters in mammary tissue has been established using isolated mammary epithelial cells (MEC) from a variety of species.^18^, ^19^, ^20^, ^21^, ^22^, ^23^, ^24^, ^25^, ^26^ One of the earlier works exploring transporters in lactating human tissue identified several drug transporters including MRP2 (*ABCC2*), OCT1 (*SLC22A1*), and OCT3 (*SLC22A3*).^20^ While BCRP was not included in the analysis, several of those transporters were detected at higher RNA levels in cells from lactating compared to nonlactating tissue, indicative of the additional cellular development that MEC undergo during pregnancy. This study also investigated RNA expression of drug transporters in lactating tissue in comparison to the typical drug eliminating organs such as the liver and kidney. Actin‐normalized RNA expression showed that lactating tissue had higher levels of PEPT2 (*SLC15A2*) and OATP1A2 (*SLCO1A2*) relative to liver and kidney, respectively; however, these trends would need to be confirmed on the protein level.

Jonker et al.^19^ specifically explored the role of BCRP in lactation and showed differential BCRP expression on the apical side of MEC between non‐lactating and lactating mouse, cow, and human mammary glands using immunohistochemistry. Apical expression of the mouse ortholog of BCRP (Abcg2) was also clearly demonstrated in wild‐type mice and was absent in Abcg2 knockouts using immunohistochemistry techniques.^21^ Western blot analysis was also performed on crude membrane fractions from mouse mammary tissue and demonstrated time‐dependent expression of Abcg2, with protein levels increasing over pregnancy, reaching a maximum during lactation and decreasing during mammary gland involution.^19^ The rat ortholog of BCRP has been identified in lactating rat mammary glands using similar analytical techniques.^22^ A difference in BCRP protein abundance between lactating and non‐lactating sheep, cows, and goats has been observed using Western blot analysis of mammary tissue, with BCRP levels approximately 10 to 21 times higher in lactating versus non‐lactating animals.^26^

BCRP has also been identified in in vitro MEC cell lines. A comprehensive analysis of HC11 mammary epithelial cells (derived from mice) examining mRNA and protein expression along with RNA interference (RNAi) experiments demonstrated that BCRP is upregulated in differentiated versus undifferentiated HC11 cells.^23^ Conversely, P‐gp was shown to be downregulated in both mice and humans.^18^, ^23^ The differential expression of BCRP versus P‐gp in MEC enables easier identification of the role of BCRP in drug distribution in milk as several drugs are substrates of both transporters.^27^ Mammary epithelial cells isolated from pigs have also showed expression of BCRP (Abcg2).^24^ BCRP expression in human MEC (isolated from milk) having been cultured for approximately 22 days was found to be lower than in freshly isolated cells.^25^

### Evidence of BCRP in lactation in animals

BCRP's role in lactational drug distribution has been studied in numerous animal models. Probably the most relevant to the pharmaceutical industry is the knockout mouse, which has been well established in elucidating the role of transporters in drug disposition. Several studies involving BCRP knockout mice have demonstrated the role of this transporter in drug, dietary component, or toxin secretion into milk, as summarized in Table 1 (drugs) and Table S1 (dietary component and toxins). Higher M:P ratios were consistently observed in Bcrp wild‐type mice compared to Bcrp knockouts, supporting the key role played by this transporter in lactational drug distribution.

**TABLE 1:** Milk:Plasma ratios of BCRP drug substrates in wild‐type and Abcg2 −/− knockout mice.

Agent investigated	Dose; Sample collection time	Milk:Plasma ratios		Reference
		WT	Abcg2 −/−	
Acyclovir	4 mg/kg/day IP; 60 h	1.5	0.1	84
Cimetidine	1 mg/kg IV; 30 min	14	2.3	19
	2.65 mg/kg/day IP; 60 h	12	2.9	84
Ciprofloxacin	10 mg/kg IV; 10 min	3.1	1.6	85
Clorsulon	5 mg/kg IV; 30 min	1.0	0.51	86
Danofloxacin	10 mg/kg IV; 20 min	5.5	3.1	87
Flunixin	4 mg/kg IV; 40 min	0.43	0.14	31
Meloxicam	10 mg/kg IV; 30 min	0.58	0.23	88
	15 mg/kg PO; 2 h	0.40	0.21	
Moxidectin	0.5 mg/kg IV; 30 min	1.0	0.6	89
	0.5 mg/kg IV; 60 min	2	1.3	
Nitrofurantoin	5 mg/kg IV; 30 min	46	0.6	90
Topotecan	1 mg/kg IV; 30 min	6.8	0.7	19

Abbreviations: IP, intraperitoneal; IV, intravenous; PO, per os (oral); WT, wildtype.

It should be noted that a common design of the knockout mouse studies was to measure the M:P ratio from a single timepoint after intravenous drug administration. Although intravenous drug administration avoids the contribution of BCRP in the gut during oral absorption, M:P ratio calculation is more appropriate from an AUC ratio which reflects the full time‐course of drug distribution in milk and plasma. The single sampling is not ideal but is understandable considering the small sample volumes obtained in mice and still demonstrates the potential for milk concentrations to be greater than plasma for known BCRP substrates.

Active transport of substrates by BCRP in the mammary gland has been shown to be inhibited by BCRP inhibitors in vivo.^19^, ^22^, ^28^, ^29^ Topotecan distribution into milk of WT mice was decreased by BCRP inhibitor GF120918 (elacridar), resulting in a M:P ratio comparable to data in BCRP knockout counterparts,^19^ while the M:P ratio in BCRP knockout mice was unaffected by co‐administration of GF120918. Similarly, coadministration of GF120918 with nitrofurantoin in lactating rats resulted in higher serum and lower milk concentrations causing milk:serum (M:S) ratios to decrease from 41.5 to 3.04 with the addition of this BCRP inhibitor.^22^ Nitrofurantoin distribution into milk was also inhibited by isoflavones genistein and daidzein and the M:P ratio decreased from 7.1 to 4.2 in WT mice, but was unaffected in BCRP KO mice.^28^ Differential effect on pantoprazole M:S ratios was noted by coadministration of GF120918 in lactating rats with (−)‐pantoprazole showing a greater decrease in M:S than (+)‐pantoprazole.^29^ In addition to studies in mice and rats, several BCRP drug–drug interaction studies have been conducted in sheep with veterinary drugs (Table S2).

Inhibition of mammary BCRP will have an impact on drug exposure to the neonate and result in lower drug exposure via milk to infants (beneficial for infant). In contrast, a potential increase in maternal systemic concentration is unlikely to be to the same extent as mammary BCRP efflux is generally not the major elimination route for a drug of interest. However, for a drug where BCRP contributes significantly to disposition (e.g., rosuvastatin) there is potential for a more sizable impact to maternal systemic concentration.^30^ Consideration for the disposition of the inhibitor should also be explored as it could distribute into milk via other pathways (passive or active). This demonstrates the complicated processes involved in lactation and that drug exposure in both the mother and infant need to be considered, particularly for drug–drug interactions.

Distribution of metabolites into milk may also be affected by BCRP.^31^, ^32^ Albendazole is an anthelmintic that is metabolized to a sulfoxide (ABZSO), sulfone (ABZSO_2_) and 2‐aminosulphone (ABZSO_2_‐NH_2_). Plasma concentrations for each metabolite were similar between WT and KO mice upon intravenous administration of each metabolite individually; however, milk concentrations were decreased in KO mice.^32^ Resulting M:P ratios were 1.13, 1.52 and 6.60 for ABZSO, ABZSO_2_ and ABZSO_2_‐NH_2_ in WT mice, respectively, compared to 0.89, 1.09 and 3.00 for ABZSO, ABZSO_2_ and ABZSO_2_‐NH_2_ in KO mice, respectively. Similarly, the M:P ratio of 5‐hydroxyflunixin, a metabolite of flunixin, was approximately 5 times higher in WT versus KO mice when administered by itself intravenously.^31^

BCRP is one of a handful of proteins involved in drug disposition that exhibit genetic polymorphisms. Y581S is a single nucleotide gain‐of‐function polymorphism specific to cows and it affects milk production and content.^31^ Western blot analysis did not detect an increase in protein expression between the polymorphisms, suggesting the gain‐of‐function was due to increased transporter capacity in this species.^33^ Cows with the Y/S heterozygous polymorphism have been shown to have higher M:P ratios compared to their Y/Y homozygous counterparts (Table S3). This is in contrast to humans which seem to display loss‐of‐function polymorphisms for BCRP^34^; however, the experiments in cows show that BCRP genetic polymorphisms can affect drug distribution into milk.

Longitudinal changes in BCRP mRNA expression have been observed in mouse mammary tissue from pregnancy through lactation.^35^ Relative BCRP gene expression increased from early gestation to late lactation with a significant drop in expression by the second day of weaning.

The above combined information obtained from in vivo animal experiments from both the pharmaceutical and veterinary field not only establish BCRP's presence in mammary tissue, but also demonstrate its influence in drug distribution in milk and the many factors affecting that process.

### Clinical evidence of BCRP in lactation

Current clinical evidence of the role of BCRP in lactational drug distribution in human is sparse due to the rarity of clinical lactation pharmacokinetic studies and the small number of subjects utilized in studies reported in literature, as summarized in Table 2. Contribution of BCRP to milk distribution of drugs that are substrates for this transporter can be inferred from comparisons of the clinically observed M:P ratio to values predicted using conventional equations based on passive permeability^14^ or inclusion of active transport via BCRP based on in vitro efflux ratios.^16^ Since the comparison is made to predicted M:P ratios, consideration should be taken for the assumptions and errors around those predictive equations.

**TABLE 2:** Observed and predicted milk:plasma ratios of BCRP substrates in humans.

Milk:Plasma (M:P) ratios						
Drug	Number of subjects	Approx. age of milk	Predicted M:P ratio passive [active]^a^	Mean observed	Method for observed M:P ratio	Reference
Acyclovir	1	4 months	0.75 [2.2]	9.54	AUC	91
	1	1 year		3.24	AUC	92
	1	7 weeks		2.25	AUC	93
Apixaban	1	23 months	0.24 [5.2]	2.61	AUC	94
Cimetidine	12	6 to 45 weeks	1.08 [3.7]	5.77 (serum)	AUC	95
	1	6 months		4.60–11.8	Individual timepoint	96
Ciprofloxacin	1	3 months	0.02 [0.86]	4.67 (serum)	Individual timepoint	97
	10	Unknown		1.82 (serum)	AUC	98
Nifedipine	1	10 days	0.28 [NA]	0.65 (serum)	AUC	99
	13	15 to 30 days		0.73 (WT)	Individual timepoint	40
	6			1.2 (Het)		
Nitrofurantoin	6	3 to 6 days	0.09 [4.4]	2.13–2.43	Individual timepoint	39
	4	8 to 26 weeks		6.19 (serum)	AUC	38
Rosuvastatin	1	13 months	0.04 [5.8]	16.5	Individual timepoint	100

Abbreviations: AUC, M:P determined from AUC ratio or ratio of average; Het, ABCG2 c.421C>A; NA, not available due to scarcity of reported Caco‐2 efflux ratio in literature; WT, wild type.

^a^ M:P ratio derived from passive permeability calculated by Atkinson & Begg equations^14^ and active transport calculated by Yang et al. equations^16^ (see Table S4 for a description of the equations and parameters used in calculations).

Because of the sparsity of clinical data, the quality of the studies that are found in literature also needs to be considered. Nitrofurantoin is an antibiotic intended to treat urinary tract infections and a known BCRP substrate. Early literature exploring nitrofurantoin distribution into milk reported a M:P ratio of approximately 0.3, if drug was detected in milk at all.^36^, ^37^ More recent literature indicates that nitrofurantoin is highly distributed into milk with a M:P ratio as high as 6 being measured.^38^ The analytical and sampling methods used in older versus newer studies need to be considered. In this particular case, the older studies used analytical methods with a high limit of quantification and there was no mention of any steps taken to preserve sample stability.^38^, ^39^ Given that some clinical lactation studies involve at‐home sample collection, it is important to understand sample stability and ensure that sample collection protocols are strictly followed.

One study reported that the M:P ratio of nifedipine is affected by BCRP polymorphism.^40^ A median M:P ratio of 0.73 was reported in wild‐type subjects (*n* = 13) compared to approximately 1.2 in heterozygous 421C>A subjects (*n* = 6). The higher observed M:P ratio in women heterozygous for 421C>A is contrary to what would be expected for this allele which demonstrates a lower BCRP expression.^41^ Nifedipine plasma concentrations were similar between the two groups; however, milk concentrations were higher in the 421C>A group compared to wild type, and highly variable. Notably, the M:P ratio was determined at trough concentrations and a single timepoint may not be reflective of the entire drug distribution time course.

Based on the overall trend of BCRP substrates in Table 2, M:P ratios are highly variable and can be drastically different from M:P ratios predicted via passive permeability (2‐ to 413‐fold higher).

### Hormonal regulation of BCRP

To understand BCRP, its role in lactation and how that may change over time, it is important to appreciate the physiological changes during pregnancy and lactation. While puberty marks the onset of breast development, that development remains incomplete until pregnancy when mammary cell maturation occurs, and breast tissue finally achieves its functional capability.^42^ Prior to pregnancy, breast tissue is primarily made up of adipose tissue compared to glandular or ductal tissue. During pregnancy, progesterone and estrogen trigger proliferation and elongation of ductal tissue, increased lobular branching, and differentiation of mammary alveoli.^42^, ^43^ Estrogen also increases the size of the anterior pituitary gland by increasing the number and size of lactotrophs, which ultimately leads to production of more prolactin.^43^ Prolactin is the main hormone responsible for lactation by stimulating MEC to make milk components. Milk can be produced by approximately 20 weeks gestation, but full production is inhibited by sex hormones. Progesterone, in particular, down‐regulates prolactin receptors on mammary alveolar cells and milk component synthesis and prevents mammary epithelial cells from forming tight junctions.^42^, ^43^ After childbirth, concentrations of those hormones decrease, especially progesterone with the delivery of the placenta, and prolactin and oxytocin increase.^43^ As such, lactogenesis during pregnancy and to approximately 2 days after birth is largely driven by paracrine hormonal regulation, while on‐going milk production is driven by autocrine regulation ultimately dictated by milk removal.^44^ During this stage suckling and the degree to which the breast is emptied control milk synthesis more so than serum prolactin levels, where no correlation was identified between the hormone and rate of milk synthesis.^42^, ^44^ When suckling stops, prolactin levels drop and tissue remodeling of the mammary tissue begins when suckling ceases for long enough.^43^

Understanding what happens to BCRP against the backdrop of these numerous biological changes in a woman's body during pregnancy and lactation is challenging. Several nuclear hormone receptors have been identified as having a potential involvement in regulating its expression including the estrogen receptor, progesterone receptor, CAR, PXR, and AHR.^45^ The higher BCRP levels noted in mature mammary glands compared to non‐lactating tissue (determined by Western blot analysis or immunohistochemical staining^19^) could be a natural state, that is, it is not an induction of transporter, as seen with P‐gp, OATP1B1 (*SLCO1B1*) and OCT2 (*SLC22A2*) during pregnancy,^46^ but a byproduct of a cell reaching full maturity. It is known that breastfeeding affects ovulation in the weeks after birth and is due to prolactin's inhibitory effects on gonadotropin‐releasing hormone which further affects levels of luteinizing hormone.^47^ If estrogen and progesterone are involved in regulating BCRP, then it is also possible that BCRP expression in lactating mothers may fluctuate when ovulation resumes.

## CURRENT STATUS OF BCRP QUANTITATIVE EXPRESSION DATA AND APPLICATION

### BCRP expression in mammary tissue and milk

Currently, there are no quantitative proteomic data on expression of BCRP in human mammary tissue over the course of lactation. Studies in animals have reported up to 21‐fold higher BCRP protein expression (based on Western blot analysis) in mammary tissue of lactating versus non‐lactating animals.^26^, ^48^

Several proteomic studies have been performed on human milk, and while BCRP may have been identified among the large datasets, few sources specifically focused on quantifying BCRP. Ahmadzai et al.^25^ quantified BCRP in human milk in a longitudinal study that included 22 women over a year of lactation, although only 10 women provided samples across the entire sampling time course. Interindividual variability was high, and it should be noted that subjects were not genotyped. BCRP mRNA quantified from cells isolated from breast milk showed a relative increase in BCRP gene expression from 1 to 5 months and a decrease from 5 to 12 months. The study did not specify if the women were exclusively breastfeeding over the sampling period, but solid foods are typically introduced into a baby's diet at about 6 months (When, What, and How to Introduce Solid Foods|Nutrition|CDC). The increase in BCRP mRNA observed over the first 5 months could reflect changes in sex hormones as ovulation resumes due to supplemented breastfeeding,^49^ assuming that estrogen and progesterone are involved in regulating BCRP expression in the breast.^45^ Subsequent decrease in BCRP gene expression could reflect the first stage of involution, which is reversible if breastfeeding is resumed,^50^ as infants start to wean.

### BCRP expression in infant gastrointestinal tract

Understanding BCRP expression and activity as it changes with time during lactation is equally important in the infant, particularly in the intestine where the first interface of a drug ingested via milk will occur. While BCRP is actively transporting a substrate into milk, BCRP will play a more protective role in the small intestine of an infant by transporting drug back into the intestinal lumen (Figure 1). Similar to other pediatric data, not a lot is definitively known about the abundance of BCRP in the infant intestine, regional differences, or its ontogeny. Several previous reviews on the topic cited the same early dataset that reported BCRP immunohistochemical staining in intestinal samples (unspecified region) from fetuses from about 6 to 28 weeks gestation,^8^, ^51^, ^52^ where BCRP staining appeared to be stable during that time frame and comparable to adults.^53^ Recent proteomic work has shed some light onto the quantitative abundance of BCRP in pediatric intestine.^54^, ^55^ However, typical with work of this nature, data are constrained by low subject numbers per age group (particularly for neonates) and the fact that tissue samples tend to be more readily available from subjects with underlying disease, which may not reflect healthy subjects. Nevertheless, existing data indicate similar longitudinal trends in abundance of intestinal BCRP in the pediatric population compared to adults when normalized to villin‐1, an age‐independent protein expressed in enterocytes (Table 3). Jejunal and ileal tissues leftover from intestinal surgeries showed that normalized BCRP protein abundance was approximately 3‐fold higher in adults than in pediatric subjects grouped from ages 0–2, 2–12 (ileum only), and 2–18 years old, while protein abundance was similar between those pediatric age groups studied.^54^ Proteomic study of duodenal pinch biopsies from children collected as part of clinical care revealed conflicting evidence in terms of BCRP protein expression due to technical challenges with the probes used for analysis.^55^ As such, a range of possible BCRP protein abundances were provided based on the probe used with a general trend showing that the lowest levels of duodenal BCRP were in the youngest subjects (<1 year). High variability was noted in both studies. In addition to proteomics, Ussing chamber experiments with ileal samples showed a higher efflux ratio for rosuvastatin in adults compared to pediatrics (2.1 vs. 1.3).^56^ Quantitative protein expression data collected thus far indicate higher BCRP abundance in adults compared to pediatrics. However, it is unclear when the increase happens, as current sparse data indicate relatively stable BCRP protein abundance up to age 18.

**TABLE 3:** Pediatric Intestinal BCRP Protein Levels (Villin‐Normalized) Determined by LC–MS/MS Reported in Literature.

Intestinal segment	Age group	*N*	Average BCRP (pmol/mg protein^a^)	Peptide	Reference
Duodenum	11 months	1^b^	1.6	VIQELGLDK	55
	2–5 years	6	3.0		
	6–12 years	9	4.9		
	12–15 years	8	4.4		
	11 months	1^b^	1.3	LFDSLTLLASGR	
	2–5 years	4	1.1		
	6–12 years	8	1.0		
	12–15 years	6	1.3		
Jejunum	0–2 years	8	32	SSLLDVLAAR	54
	2–18 years	2	34^c^		
	Adults	8	92		
Ileum	0–2 years	29	32		
	2–12 years	6	41^c^		
	12–18 years	13	29		
	Adult	8	102		

^a^ Refers to mg of homogenate protein.

^b^ Infant diagnosed with Celiac's disease.

^c^ Determined from data image capture using (WebPlotDigitizer automeris.io). Remaining data are from text or supplementary tables.

### Prediction methods for BCRP in lactation

As previously mentioned, M:P ratios for a drug can be predicted by taking into account drug physicochemical properties and composition of milk.^14^ Attempts have been made to improve upon the existing physicochemistry‐based predictions by incorporating active transport of BCRP and P‐gp substrates. Ito et al.^15^ developed a linear relationship between in vitro generated BCRP efflux ratios from MDCK II‐BCRP cells and the ratio of observed versus predicted unbound M:P ratios. By incorporating a BCRP scaling factor determined from this linear relationship, they improved predictions of M:P ratios for BCRP substrates (*r* ^2^ of 0.091 based on passive permeability alone vs. 0.89 with the new equation). However, this work utilized a small subset of drugs to develop the mathematical relationship (five in development set; eight in validation set). A similar approach was developed using the efflux ratio obtained from in vitro permeability data from Caco‐2 cells with the aim to refine predictions of M:P ratios.^16^ Geometric mean fold error in predicted M:P ratio was improved using this approach, but the analysis equally included a small dataset of BCRP substrates (*n* = 5). While M:P ratio predictions for BCRP substrates were improved by efflux ratio data obtained in standard cell lines used in permeability assays, the approaches described are still empirical and do not account for expression or proteomic differences in the cell lines versus mammary tissue which is an essential element of any reliable transporter IVIVE.^57^ Improvement of IVIVE approaches to better predict distribution of drugs into milk and M:P ratios of BCRP substrates could ideally consider using a cell line more relevant to mammary gland tissue. However, it is appreciated that more investigation is needed to find an optimal mammary epithelial cell line and experimental conditions,^18^ not to mention the feasibility of including such an experiment in a typical drug discovery or development program where Caco‐2 and MDCK experiments are routinely conducted.

PBPK modeling has been successfully used to predict enzyme and transporter‐mediated processes in other special populations.^8^, ^9^, ^58^ A comprehensive review of the current status of PBPK models in lactation was recently published.^10^ One shortcoming noted in Lact‐PBPK models is the lack of mechanistic models of breast tissue and milk secretion linked to mechanistic oral absorption models of infant which captures the numerous short‐term and long‐term physiological dynamics observed during weaning (e.g., postpartum changes in the mother and ontogeny in the infant). Additionally, there is limited translational understanding of lactation across species, a lack of lactation models for large molecules (e.g., biologics), and many Lact‐PBPK models generally describe passively permeable drugs, with limited inclusion of drugs that are actively transported. A separate search revealed that of 74 articles describing Lact‐PBPK only one attempted to include BCRP.^59^ Zhang et al.^59^ used intrinsic clearance of 5 BCRP substrates (obtained from measured *V*_max_ and *K*_m_) and used a relative expression factor (REF) to scale BCRP intrinsic clearance in mammary tissue, accounting also for an estimated mammary alveolar surface area. Differences in BCRP protein expression (determined by Western blot analysis) and mRNA expression in mammary tissue of lactating and non‐lactating animals was used to inform the REF. BCRP protein expression was found to be approximately 10‐ to 21‐fold higher in lactating versus non‐lactating animals (cows, sheep, and goats), which was similar to the mRNA analysis (15‐fold increase in mice). Predicted M:P ratios (calculated from AUC data) were within 1.5‐fold of observed for drugs investigated indicating the potential feasibility of using animal data to inform a human model. However, initial BCRP clearance values were optimized during modeling using the same clinical datasets used for model verification.

The lack of examples of transporter‐based Lact‐PBPK is in large part due to the absence of longitudinal transporter expression or activity data and appropriate scaling factors needed to fully inform and implement transporter dynamics in these models. Capturing the dynamics of the volume of breast milk within the breast, volumes consumed by an infant during a feeding and system parameters describing the ontogeny of proteins that affect drug absorption, distribution and metabolism in the infant from birth to weaning further complicate modeling efforts to more accurately reflect the ever‐changing biology involved in lactation. While model simplification with assumptions that allow for conservative risk assessment is certainly justified, more dynamic models may demonstrate other opportunities for breast feeding without sacrificing drug efficacy or safety.

## BRIDGING THE BCRP GAPS

As indicated in the previous section, limited expression and functional data on BCRP in human mammary tissue and its ontogeny in infants are available and several gaps remain. Sample availability remains an obstacle.^60^ Ideally, samples should be collected from the same individual over time (weeks/months for lactating women and months/years for infants). However, collecting mammary tissue from healthy lactating women and intestinal segments from healthy infants is not only unlikely, but potentially also raises ethical concerns. If direct measures in tissue are not practical, then alternative means must be considered. Measures of drug concentration in milk itself provide a non‐invasive approach to understanding potential physiological changes in mammary tissue. However, it limits analysis to women who are actively lactating (vs. non‐lactating) and still relies on a subject's ability to collect, store and ship samples per protocol. Barring these typical concerns any clinical study face, several approaches are available to explore not only BCRP, but any ADME‐related enzyme or transporter.

Recent work with liquid biopsies and isolation of exosomes has been successfully implemented in understanding changes in expression of certain hepatic enzymes and transporters (e.g., CYP3A4, OATP1B1, P‐gp, and BCRP) in different populations.^61^, ^62^ Currently, only liver specific small extracellular vesicles/exosomes have been isolated, as tissue‐specific markers have not been validated for other tissues. However, studies so far highlight a potential avenue of analysis to understand BCRP expression quantitatively in mammary tissue.^63^ While obtaining information on protein or mRNA expression in tissues from plasma exosomal samples is non‐invasive and avoids complications associated with a tissue biopsy, the technique is still developing. One potential concern is that the amount of protein isolated from mammary‐derived exosomes in plasma may be below quantification limits.^64^

Since milk comes directly from mammary tissue, direct proteomic analysis of milk following trypsin digestion is another possibility. Methodology adapted from Vincent et al.^65^ is currently being used in our laboratories to identify BCRP presence in rat and human milk. It is important to note that liquid biopsies and proteomics only provide data on protein expression or changes in expression within a population. Further data and interpretation are needed to link protein or mRNA expression to functional activity of an enzyme or transporter.^66^

In addition, endogenous biomarkers have been successfully used to explore potential changes in transporter‐mediated drug disposition as a result of transporter‐mediated interactions or in certain disease populations.^67^ For example, coproporphyrin‐I has been used as a biomarker for OATP1B1 (*SLCO1B1*) to evaluate drug–drug interaction risk early in clinical development.^68^, ^69^ In contrast to OATP1B1, the number of possible biomarkers of BCRP is limited. One potential biomarker proposed to evaluate changes in BCRP activity is riboflavin (vitamin B2). Early work demonstrated a 22‐fold decrease in riboflavin M:P ratio in BCRP knockout mice after riboflavin was administered intravenously compared to wild‐type mice.^70^ In contrast, no significant difference in riboflavin milk concentrations were observed in the milk from heterozygous cows with the Y581S gain‐of‐function allele compared to wild‐type cows.^71^ It should be noted that only milk concentrations were reported in this study. Analysis of plasma would have provided more insight whether riboflavin's M:P ratio was different in cows with and without the polymorphism as riboflavin is obtained either in diet or by gut microflora in this species.^72^ Considering that BCRP is also located at the intestinal interface, plasma levels would be expected to differ in wild‐type cows versus those with the Y581S allele. Indeed, lower plasma levels of BCRP substrates were noted in several studies in cows heterozygous for the Y581S allele which would result in a higher M:P ratio in these animals (Table S3). More recent exploration of riboflavin in BCRP and P‐gp knockout mice has shown its specificity for BCRP, with no overlap with P‐gp.^73^ In addition, riboflavin AUC ratios correlated with AUC ratios of BCRP substrate sulfasalazine in a monkey drug–drug‐interaction study in the presence of BCRP inhibitor ML753286, highlighting its potential as a BCRP endogenous biomarker.^73^

A potential challenge in exploring riboflavin as a biomarker for monitoring BCRP in vivo is that riboflavin is a substrate of additional transporters. Riboflavin uptake is mediated by active transport via three riboflavin transporters (RFVT1, RFVT2, and RFVT3 (*SLC52A1–3*)), which is saturable.^73^, ^74^ Once absorbed, riboflavin is primarily eliminated via urine with evidence of both tubular secretion and reabsorption,^75^, ^76^ and it has recently been identified as an in vitro substrate of OAT1 (*SLC22A6*), OAT3 (*SLC22A8*), and MATE2‐K (*SLC47A2*) transporters.^77^

Another challenge is that riboflavin is not synthesized by humans, rather it is obtained from the diet or synthesis by gut microflora.^74^ Despite the lack of a steady synthesis source, studies have shown that riboflavin levels in plasma in an individual are relatively resistant to circadian rhythms or diet.^73^, ^75^ Riboflavin levels in milk, however, have been shown to increase with vitamin supplementation.^78^, ^79^ While low intra‐individual variability in riboflavin baseline points to riboflavin being tightly controlled within an individual, high inter‐individual variability has been noted in plasma with coefficients of variation of approximately 50%–60%.^73^

Another approach to understand the role of BCRP is to consider tool compound(s), where changes in clearance for a specific substrate of a drug metabolizing enzyme/transporter in a special population compared to a healthy population are used to inform potential changes in the activity of that protein of interest for another substrate. This concept has been applied to evaluate changes in OAT1/3 activity in patients with renal impairment^80^ or OATP1B1 in hepatic impairment.^81^ This approach has been successfully utilized in pregnancy PBPK modeling^82^ and could be useful in determining BCRP's role in lactation. This approach requires careful consideration of all other potential physiologic changes in the special population that may affect the pharmacokinetics of selected probe(s), like changes in protein binding, so that they do not confound the final assessment of the enzyme or transporter of interest.^8^, ^80^, ^81^

The section above clearly illustrated that no single approach to quantitative understanding of BCRP's role in lactation is perfect and multiple, integrated approaches should be utilized in tandem so that the totality of the data improves our understanding of BCRP in lactation and drives successful implementation in a Lact‐PBPK model.

## CONCLUDING REMARKS

Several gaps in knowledge exist in the lactation space, not only for BCRP, but also for other transporters and drug‐metabolizing enzymes that may be expressed in mammary tissue.^17^ Understanding longitudinal changes in BCRP expression is important to deduce if there are time‐related changes in drug disposition that could inform infant risk assessments, especially in early (few days after birth where drastic hormone changes occur and lactation starts), mid‐ (where lactating women experience amenorrhea) and late lactation (where infants have started to wean). Further, understanding the drivers of BCRP regulation, and how they relate to sex hormone changes postpartum, would be helpful in contextualizing any BCRP abundance or activity data. It is also important to consider that any pre‐existing or acute disease in lactating women could further affect drug disposition by altering expression of BCRP or other transporters or by changing the physiology of the tissue itself which could affect a drug's passive permeability. Inflammation may be a potential mediator in transporter expression in MEC, as evidenced by recent in vitro studies in mouse mammary epithelial cells treated with lipopolysaccharide.^83^

While the focus is on informing human lactation and drug labels, time should be spent on understanding lactation in typical preclinical species used in reproductive toxicology assessment to allow better extrapolation to human. It is understood that the physiology and even milk content varies between mammalian species,^12^ however, quantitative understanding of those differences can allow extrapolation to human by identifying common trends in drug behavior, similar to how preclinical data are utilized in first‐in‐human dose predictions particularly with PBPK modeling. This will allow better implementation of data that may already exist in a pharmaceutical company's repository.

A recent report by Grillo et al.^7^ was a clear example of how the mechanistic PBPK models, which were originally used to make dosage recommendations in specific conditions (drug–drug interactions in renally impaired patients in the absence of dedicated studies due to practical and ethical reasons), could be complemented by post‐marketing indirect evidence. The philosophy is not specific to a certain area and may cover other similar situations where real‐world data can be used to back up the initial mechanistic models. This approach has opened a window of opportunity for more informative drug labeling in relation to exposure of infants to drugs through drug excretion in breast milk based on information on the drug obtained by conducting in vitro studies.

It is appreciated that BCRP is only one transporter among many expressed in mammary tissue. Ideally, full quantitative expression data and understanding of the longitudinal changes, if any, of transporters and drug metabolizing enzymes in mammary tissue and in the infant is needed to enable mechanistic quantitative modeling, like PBPK, to reach its full potential. Quantitative understanding of key ADME‐related metabolizing enzymes and transporters in breast tissue will allow mechanistic modeling approaches to potentially replace clinical studies similar to modeling prediction successes seen for drug–drug interactions.

## FUNDING INFORMATION

No funding was received for this work.

## CONFLICT OF INTEREST STATEMENT

C.S., H.S., Y.Z., M.S., B.J.S., and L.G. are employees of and own stock in Bristol Myers Squibb. A.R.H. and A.G. research is sponsored by a consortium of pharmaceutical companies within the Centre for Applied Pharmacokinetic Research. A.R.H. holds stocks of Certara which provides modeling and simulation platforms to academic and industrial institutions.

## Supporting information


Tables S1‐S4.
