# Supplementary material for: A close examination of BCRP's role in lactation and methods for predicting drug distribution into milk
Source: CPT Pharmacometrics Syst Pharmacol. 2024 Sep 18;13(11):1856–69. doi: 10.1002/psp4.13243 (PMC11578132; doi:10.1002/psp4.13243)
Supplement: Supplementary file 1 — Tables S1‐S4. [file PSP4-13-1856-s001.docx]

**Supplementary Material**

**A Close Examination of BCRP’s Role in Lactation and Methods for Predicting Drug Distribution into Milk**

Caroline Sychterz^1,2^ | Hong Shen^2^ | Yueping Zhang^2^ | Michael Sinz^2^ | Amin Rostami-Hodjegan^1,3^ | Brian J. Schmidt^2^ | Lu Gaohua^2^ | Aleksandra Galetin^1^

^1^Centre for Applied Pharmacokinetic Research, Division of Pharmacy and Optometry, School of Health Sciences, University of Manchester, Manchester, UK

^2^Bristol Myers Squibb, Princeton, New Jersey, USA

^3^ Certara Predictive Technologies, Certara UK, Sheffield, UK

**Supplementary Table 1: Milk:Plasma Ratios of Additional BCRP Substrates in Wild Type and Abcg2 -/- Knockout Mice (Dietary Components and Toxins)**

| Agent Investigated | Dose;  Sample Collection Time | Milk:Plasma Ratios | | Reference |
| --- | --- | --- | --- | --- |
|  |  | WT | Abcg2 -/- |  |
| 2-amino-3-methylimidazo[4,5-f]quinoline | 1 mg/kg IV; 30 min | 0.9 | 0.3 | ^1^ |
| 3-amino-1,4-dimethyl-5H-pyrido[4,3-b]indole | 1 mg/kg IV; 30 min | 1.2 | 0.4 | ^1^ |
| Aflatoxin B1 | 1 mg/kg IV; 30 min | 0.7 | 0.2 | ^1^ |
| Enterolactone | Dietary plant lignan | 6.4 | 0.4 | ^2^ |
| Riboflavin | 1.67 nmol/kg IV; 30 min | 9.9 | 0.44 | ^3^ |
| Tryptophan | Amino acid and metabolites | 0.004 | 0.002 | ^4^ |
| Kynurenine |  | 0.02 | 0.01 |  |
| Kynurenic acid |  | 6.9 | 1.5 |  |
| Xanthurenic acid |  | 0.97 | 0.22 |  |
| Anthranilic acid |  | 0.23 | 0.12 |  |

**Supplementary Table 2: Milk:Plasma Ratios of BCRP Substrates from Drug-Drug Interaction Studies in Animals Not Discussed in Text**

| Agent Investigated | Inhibitor | Species | Milk:Plasma Ratios | | Reference |
| --- | --- | --- | --- | --- | --- |
|  |  |  | Control | With Inhibitor |  |
| Clorsulon | Ivermectin | Mice | 1.01 | 0.43 | ^5^ |
| Danofloxacin | Flavonoids (soy-enriched diet) | Sheep | 9.58 | 4.90 | ^6^ |
|  | Ivermectin | Sheep | 9.58 | 5.74 | ^7^ |
|  | Triclabendazole | Sheep | No effect | | ^8^ |
|  | Eprinomectin | Sheep | No significant effect although plasma AUC increased | | ^9^ |
| Enrofloxacin | Genistein | Sheep | No significant effect although milk AUC decreased | | ^10^ |
|  | Albendazole sulfoxide | Sheep | No significant effect although milk AUC decreased | | ^10^ |
| Meloxicam | Ivermectin | Sheep | 0.17 | 0.09 | ^11^ |
|  | Eprinomectin | Sheep | No significant effect although plasma AUC increased | | ^9^ |
| Montepantel sulfone | Isoflavones | Sheep | 5.46 | 3.27 | ^12^ |
| Moxidectin | Triclabendazole | Sheep | 11.68 | 7.34 | ^8^ |
| Nitrofurantoin | Triclabendazole sulfoxide (metabolite of triclabendazole) | Mice | 2.80 a | 0.93 (50 mg/kg) a  0.76 (100 mg/kg) a | ^13^ |
|  | Isoflavones | Sheep | Highly variable data | | ^14^ |

a Determined from data image capture using (WebPlotDigitizer [automeris.io](https://automeris.io/WebPlotDigitizer.html)). Remaining data are from text or supplementary tables

**Supplementary Table 3: Milk:Plasma Ratios of BCRP Substrates from BCRP Polymorphism Studies in Cows**

| Agent Investigated | Milk:Plasma Ratio | | Other Observations | References |
| --- | --- | --- | --- | --- |
|  | Y/Y581 homozygous | Y/S581 heterozygous |  |  |
| Ciprofloxacin | 3.61 | 10.19 | Lower plasma levels and higher milk levels in Y/S581 heterozygous | ^15^ |
| Danofloxacin | 4.09 | 8.81 | Plasma levels similar between groups; higher milk levels in Y/S581 heterozygous | ^16^ |
|  | 3.42 | 7.02 | Slightly lower plasma levels and higher milk levels in Y/S581 heterozygous | ^17^ |
| Enrofloxacin | 0.56 | 0.81 | Lower plasma levels and higher milk levels in Y/S581 heterozygous | ^15^ |
| Enterolactone | 1.44 | 2.99 | Lower plasma levels in Y/S581 heterozygous; milk levels similar between groups | ^18^ |

| Agent Investigated | Milk:Plasma Ratio | | Other Observations | References |
| --- | --- | --- | --- | --- |
|  | Y/Y581 homozygous | Y/S581 heterozygous |  |  |
| Flunixin | 0.007 | 0.009 | Plasma levels similar between groups; higher milk levels in Y/S581 heterozygous | ^19^ |
| 5-hydroxyflunixin | 0.450 | 0.684 |  |  |
| Kynurenine | 0.02 | 0.04 | Plasma levels similar between groups; higher milk levels in Y/S581 heterozygous | ^4^ |
| Riboflavin | Not calculated; only milk reported | | No statistically significant difference in milk levels | ^18^ |
| Uric acid | 1.86 | 3.09 | Plasma levels similar between groups; higher milk levels in Y/S581 heterozygous | ^18^ |

**Supplementary Table 4: Parameters Used to Calculate Predicted M:P Ratio Based on Passive Permeability and Transporter Efflux in Table 2**

| Drug | Physicochemical Properties^a^ | | | | | M:P Ratio Predicted from Passive Permeability via Atkinson & Begg^g^ | | | | M:P Ratio Predicted from BCRP Active Transport via Yang et al.^h^ | | | |
| --- | --- | --- | --- | --- | --- | --- | --- | --- | --- | --- | --- | --- | --- |
|  | $\boldsymbol{logP}$ | $\boldsymbol{pKa}$ | $\boldsymbol{PSA}$ | $\boldsymbol{f}\boldsymbol{u}_{\boldsymbol{p}}$ | $\boldsymbol{ER}$ | $\boldsymbol{f}\boldsymbol{u}_{\boldsymbol{skim}}$ | $\frac{\boldsymbol{M}_{\boldsymbol{u}}}{\boldsymbol{P}_{\boldsymbol{u}}}$ | $\boldsymbol{D}_{\boldsymbol{pH}\boldsymbol{7.2}}^{\boldsymbol{milkfat}}$ | M:P Ratio | $\boldsymbol{F}_{\boldsymbol{ni}\boldsymbol{7.0}}$ | $\boldsymbol{F}_{\boldsymbol{ni}\boldsymbol{7.4}}$ | $\boldsymbol{f}\boldsymbol{u}_{\boldsymbol{m}}$ | M:P Ratio |
| Acyclovir | -1.56 | 9.25 A | 115 | 0.790 | 2.66 | 0.959 | 0.995 | 0.00127 | 0.753 | 0.994 | 0.986 | 0.941 | 2.21 |
| Apixaban | 1.65 ^e^ | N | 111 ^b^ | 0.13^e^ | 24 ^f^ | 0.912 | 1.00 | 17.7 | 0.240 | 1.00 | 1.00 | 0.599 | 5.21 |
| Cimetidine | 1 | 6.8 B | 114 | 0.830 | 3.23 | 0.960 | 1.12 | 1.67 | 1.08 | 0.613 | 0.799 | 0.953 | 3.66 |
| Ciprofloxacin | 1.31 | 6.09 A | 72.9 | 0.600 | 2.76 | 0.954 | 0.648 | 0.216 | 0.0161 | 0.110 | 0.0467 | 0.827 | 0.855 |
| Nifedipine | 2.69 ^c^ | 2.39 B ^c^ | 110 ^b^ | 0.04 ^c^ | NA | 0.860 | 1.00 | 389 | 0.276 | 1.00 | 1.00 | 0.518 | NA |
| Nitrofurantoin | -0.1 | 7.2 A | 121 | 0.600 | 10.1 | 0.954 | 0.774 | 0.0401 | 0.0856 | 0.613 | 0.387 | 0.878 | 4.35 |
| Rosuvastatin | 2.4 ^c^ | 4.27 A ^c^ | 149 ^b^ | 0.107 ^c^ | 83 ^d^ | 0.905 | 0.631 | 0.0303 | 0.0386 | 0.00186 | 0.000741 | 0.610 | 5.80 |

Abbreviations: A, acidic; B, basic; N, neutral; NA, not available

^a^ Physicochemical properties were collated from ^20^ unless otherwise stated. Data reported as 3-significant digits unless the original data source reported a value with less. In those cases, the values as reported were used as is.

^b^ [PubChem (nih.gov)](https://pubchem.ncbi.nlm.nih.gov/)

^c^ Simcyp v22 software compound files

^d^ [Certara Drug Interaction Solutions](https://www.druginteractionsolutions.org/)

^e^ Measured

^f^ From ^21^

^g^ Atkinson & Begg M:P ratio equations for the log phase distribution model^22, 23^:

$$\ln\left( M:P \right)=a+b*\ln\left( fu_{p} \right)+c*\ln\left( \frac{M_{u}}{P_{u}} \right)+d*ln\left( \frac{0.955}{fu_{skim}}+0.045*D_{pH7.2}^{milkfat} \right)$$

where a = -0.405, b = -0.69, c = 9.36 and d= -1.54 for acids, and a = -0.09, b = 0.79, c = 2.54 and d = 0.46 for bases. The values of 0.955 and 0.045 represent the general volume fractions of aqueous and fat portions of milk, respectively. $fu_{p}$ is the unbound fraction of drug in plasma, $\frac{M_{u}}{P_{u}}$ is the milk:plasma ratio of unbound, unionized drug determined from the Henderson-Hasselbalch equation and assuming milk has a pH of 7.2 and plasma has a pH of 7.4, $fu_{skim}$ is the unbound fraction of drug in the protein containing fraction of milk (i.e., skim milk) determined from the following equation^24^:

$$fu_{skim}=\frac{fu_{p}^{0.448}}{\left( 6.94x{10}^{-4} \right)^{0.448}+fu_{p}^{0.448}}$$

$D_{pH7.2}^{milkfat}$ is drug partitioning into milk fat at an assumed milk pH of 7.2 and is determined from the following equation^25^:

$$logD_{pH7.2}^{milkfat}=-0.88+1.29*logD_{pH7.2}$$

where $logD_{pH7.2}$ is the log of the oil to aqueous buffer ratio at pH 7.2 and can be derived from $logP_{o:w}$ and $pKa$. Note that the $logD_{pH7.2}^{milkfat}$ value needs to be converted to $D_{pH7.2}^{milkfat}$ by taking the antilog prior to use in the Atkinson & Begg equation or under-predictions in M:P ratio can occur for drugs with a high lipophilicity^26^.

^h^ Yang et al. M:P ratio equations^20^:

$$M:P=ER*\frac{fu_{p}*F_{ni7.4}}{fu_{m}*F_{ni7.0}}$$

where $ER$ is the efflux ratio determined from *in vitro* Caco-2 experiments, $F_{ni7.0}$ and $F_{ni7.4}$ are the fraction of unionized drug at pH 7.0 and 7.4 determined from the Henderson-Hasselbalch equation, respectively, and $fu_{m}$ is the unbound fraction in milk determined from the following equation:

$$f_{um}=1.033*\frac{fu_{p}}{0.988+fu_{p}}+0.1017*ln(PSA)$$

where PSA is the polar surface area of the drug.

REFERENCES

(1) van Herwaarden, A.E., Wagenaar, E., Karnekamp, B. *et al.* Breast cancer resistance protein (Bcrp1/Abcg2) reduces systemic exposure of the dietary carcinogens aflatoxin B1, IQ and Trp-P-1 but also mediates their secretion into breast milk. *Carcinogenesis.* 2006;27:123-130.

(2) Miguel, V., Otero, J.A., Garcia-Villalba, R. *et al.* Role of ABCG2 in transport of the mammalian lignan enterolactone and its secretion into milk in Abcg2 knockout mice. *Drug Metab Dispos.* 2014;42:943-946.

(3) van Herwaarden, A.E., Wagenaar, E., Merino, G. *et al.* Multidrug transporter ABCG2/breast cancer resistance protein secretes riboflavin (vitamin B2) into milk. *Mol Cell Biol.* 2007;27:1247-1253.

(4) Garcia-Lino, A.M., Gomez-Gomez, A., Garcia-Mateos, D. *et al.* Analysis of the interaction between tryptophan-related compounds and ATP-binding cassette transporter G2 (ABCG2) using targeted metabolomics. *Food Chem.* 2021;344:128665.

(5) Blanco-Paniagua, E., Álvarez-Fernández, L., Rodríguez-Alonso, A. *et al.* Role of the Abcg2 Transporter in Secretion into Milk of the Anthelmintic Clorsulon: Interaction with Ivermectin. *Antimicrob Agents Chemother.* 2023;e0009523.

(6) Perez, M., Otero, J.A., Barrera, B. *et al.* Inhibition of ABCG2/BCRP transporter by soy isoflavones genistein and daidzein: effect on plasma and milk levels of danofloxacin in sheep. *Vet J.* 2013;196:203-208.

(7) Real, R., Egido, E., Perez, M. *et al.* Involvement of breast cancer resistance protein (BCRP/ABCG2) in the secretion of danofloxacin into milk: interaction with ivermectin. *J Vet Pharmacol Ther.* 2011;34:313-321.

(8) Barrera, B., Gonzalez-Lobato, L., Otero, J.A. *et al.* Effects of triclabendazole on secretion of danofloxacin and moxidectin into the milk of sheep: role of triclabendazole metabolites as inhibitors of the ruminant ABCG2 transporter. *Vet J.* 2013;198:429-436.

(9) Garcia-Lino, A.M., Garcia-Mateos, D., Alvarez-Fernandez, I. *et al.* Role of eprinomectin as inhibitor of the ruminant ABCG2 transporter: Effects on plasma distribution of danofloxacin and meloxicam in sheep. *Res Vet Sci.* 2021;136:478-483.

(10) Pulido, M.M., Molina, A.J., Merino, G. *et al.* Interaction of enrofloxacin with breast cancer resistance protein (BCRP/ABCG2): influence of flavonoids and role in milk secretion in sheep. *J Vet Pharmacol Ther.* 2006;29:279-287.

(11) Blanco-Paniagua, E., Garcia-Lino, A.M., Alvarez-Fernandez, L., Alvarez, A.I. & Merino, G. Ivermectin inhibits ovine ABCG2-mediated in vitro transport of meloxicam and reduces its secretion into milk in sheep. *Res Vet Sci.* 2022;153:88-91.

(12) Gunes, Y., Okyar, A., Krajcsi, P., Fekete, Z. & Ustuner, O. Modulation of monepantel secretion into milk by soy isoflavones. *J Vet Pharmacol Ther.* 2023;46:185-194.

(13) Barrera, B., Otero, J.A., Egido, E. *et al.* The anthelmintic triclabendazole and its metabolites inhibit the membrane transporter ABCG2/BCRP. *Antimicrob Agents Chemother.* 2012;56:3535-3543.

(14) Perez, M., Real, R., Mendoza, G. *et al.* Milk secretion of nitrofurantoin, as a specific BCRP/ABCG2 substrate, in assaf sheep: modulation by isoflavones. *J Vet Pharmacol Ther.* 2009;32:498-502.

(15) Otero, J.A., Garcia-Mateos, D., de la Fuente, A. *et al.* Effect of bovine ABCG2 Y581S polymorphism on concentrations in milk of enrofloxacin and its active metabolite ciprofloxacin. *J Dairy Sci.* 2016;99:5731-5738.

(16) Otero, J.A., Real, R., de la Fuente, A. *et al.* The bovine ATP-binding cassette transporter ABCG2 Tyr581Ser single-nucleotide polymorphism increases milk secretion of the fluoroquinolone danofloxacin. *Drug Metab Dispos.* 2013;41:546-549.

(17) Otero, J.A., Barrera, B., de la Fuente, A. *et al.* Short communication: The gain-of-function Y581S polymorphism of the ABCG2 transporter increases secretion into milk of danofloxacin at the therapeutic dose for mastitis treatment. *J Dairy Sci.* 2015;98:312-317.

(18) Otero, J.A., Miguel, V., González-Lobato, L. *et al.* Effect of bovine ABCG2 polymorphism Y581S SNP on secretion into milk of enterolactone, riboflavin and uric acid. *Animal.* 2016;10:238-247.

(19) Garcia-Mateos, D., Garcia-Lino, A.M., Alvarez-Fernandez, I. *et al.* Role of ABCG2 in Secretion into Milk of the Anti-Inflammatory Flunixin and Its Main Metabolite: In Vitro-In Vivo Correlation in Mice and Cows. *Drug Metab Dispos.* 2019;47:516-524.

(20) Yang, H., Xue, I., Gu, Q. *et al.* Developing an In Vitro to In Vivo Extrapolation (IVIVE) Model to Predict Human Milk-to-Plasma Drug Concentration Ratios. *Mol Pharm.* 2022;19:2506-2517.

(21) Zhang, D., He, K., Herbst, J.J. *et al.* Characterization of Efflux Transporters Involved in Distribution and Disposition of Apixaban. *Drug Metabolism and Disposition.* 2013;41:827-835.

(22) Atkinson, H.C. & Begg, E.J. Prediction of drug distribution into human milk from physicochemical characteristics. *Clin Pharmacokinet.* 1990;18:151-167.

(23) Begg, E.J., Atkinson, H.C. & Duffull, S.B. Prospective evaluation of a model for the prediction of milk:plasma drug concentrations from physicochemical characteristics. *Br J Clin Pharmacol.* 1992;33:501-505.

(24) Atkinson, H.C. & Begg, E.J. Prediction of drug concentrations in human skim milk from plasma protein binding and acid-base characteristics. *Br J Clin Pharmacol.* 1988;25:495-503.

(25) Atkinson, H.C. & Begg, E.J. Relationship between human milk lipid-ultrafiltrate and octanol-water partition coefficients. *J Pharm Sci.* 1988;77:796-798.

(26) Zhang, M., Sychterz, C., Chang, M. *et al.* A perspective on the current use of the phase distribution model for predicting milk-to-plasma drug concentration ratio. *CPT Pharmacometrics Syst Pharmacol.* 2022;11:1547-1551.
